# Obese with higher FNDC5/Irisin levels have a better metabolic profile, lower lipopolysaccharide levels and type 2 diabetes risk

**DOI:** 10.1590/2359-3997000000305

**Published:** 2017-12-01

**Authors:** Ivan Luiz Padilha Bonfante, Mara Patricia Traina Chacon-Mikahil, Diego Trevisan Brunelli, Arthur Fernandes Gáspari, Renata Garbellini Duft, Alexandre Gabarra Oliveira, Tiago Gomes Araujo, Mario Jose Abdalla Saad, Cláudia Regina Cavaglieri

**Affiliations:** 1 Universidade Estadual de Campinas Faculdade de Educação Física Laboratório de Fisiologia do Exercício Campinas SP Brasil Laboratório de Fisiologia do Exercício, Faculdade de Educação Física, Universidade Estadual de Campinas (Unicamp), Campinas, SP, Brasil; 2 Universidade Estadual Paulista Instituto de Biociências Rio Claro SP Brasil Instituto de Biociências, Universidade Estadual Paulista “Júlio de Mesquita Filho” (Unesp), Rio Claro, SP, Brasil; 3 Universidade Estadual de Campinas Escola de Ciências Médicas Departamento de Medicina Interna Campinas SR Brasil Departamento de Medicina Interna, Escola de Ciências Médicas, Universidade Estadual de Campinas (Unicamp), Campinas, SR Brasil

**Keywords:** Irisin, metabolism, obese, type 2 diabetes, lipopolysaccharide

## Abstract

**Objective:**

Thus, the aim of this study was to compare if higher or smaller fibronectin type 3 domain-containing protein 5 (FNDC5)/irisin levels are associated with inflammatory and metabolic markers, caloric/macronutrient intake, physical fitness and type 2 diabetes mellitus (T2DM) risk in obese middle-aged men, and also to correlate all variables analyzed with FNDC5/irisin.

**Subjects and methods:**

On the basis of a cluster study, middle-aged obese men (IMC: 31.01 ± 1.64 kg/m^2^) were divided into groups of higher and smaller levels of FNDC5/irisin. The levels of leptin, resistin, adiponectin, tumor necrosis factor alpha (TNFα), interleukin 6 and 10 (IL6, IL10), lipopolysaccharide (LPS), glucose, insulin, glycated hemoglobin, insulin resistance and sensibility, lipid profile, risk of T2DM development, body composition, rest energy expenditure, caloric/macronutrient intake and physical fitness were measured.

**Results:**

The higher FNDC5/ irisin group presented improved insulin sensibility (homeostasis model assessment - sensibility (HOMA-S) (p = 0.01) and QUICKI index (p < 0.01)), insulin (p = 0.02) and triglyceride levels (p = 0.01), lower insulin resistance (homeostasis model assessment - insulin resistance (HOMA-IR) (p = 0.01), triglycerides/glucose (TYG index) (p = 0.02), neck circumference (p = 0.02), risk of T2DM development (p = 0.02), tendency to decrease serum resistin (p = 0.08) and significant lower LPS levels (p = 0.02). Inverse correlations between FNDC5/irisin and body weight (r −0.46, p = 0.04), neck circumference (r −0.51, p = 0.02), free fat mass (r −0.49, p = 0.02), triglycerides (r −0.43, p = 0.05) and risk of developing T2DM (r −0.61, p = 0.04) were observed.

**Conclusions:**

These results suggest that higher FNDC5/irisin levels in obese middle-aged men are related to a better metabolic profile and lower risk of T2DM development and serum LPS, a potential inducer of insulin resistance.

## INTRODUCTION

Irisin is a peptide secreted mainly by adipose tissue and muscles after the stimulation of peroxisome proliferator activates receptor gamma coactivator 1 alpha (PGC1alpha) and subsequent secretion and cleavage of fibronectin type 3 domain-containing protein 5 (FNDC5) by stimulus such as physical exercise and exposure to cold (1). In adipose tissue, especially in inguinal fat cells, irisin increases the expression of mitochondrial uncoupling protein 1 (UCP1) and, consequently, the energy expenditure and consumption of lipid reserves, which could contribute to treatment and prevention of metabolic diseases (1). Indeed, such irisin effect in inguinal seems to increase a special kind of adipocyte cell named brite or beige that show characteristics of white (basal) and brown adipocytes (after irisin stimulus) (2). Several factors are related interfering in FNDC5/irisin levels, as body composition, cold exposure, physical exercise, physical fitness and leptin levels (3-5).

The optimal levels of circulating FNDC5/irisin in humans are not established, however, some evidence indicates that individuals with type 2 diabetes mellitus (T2DM) (6,7) and metabolic syndrome and insulin resistance (IR) (8) have lower levels of this peptide, indicating that a larger amount of FNDC5/irisin could be a protective factor against these diseases. Paradoxically, some studies have shown that the presence of these diseases positively correlates with high levels of FNDC/irisin (9-11). Thus, such topic is still controversial and need to be further carefully investigated.

It is well known that there is an important imbalance in the secretion of cytokines in obese individuals, which increases the risk of developing metabolic disorders and diseases (12). Cytokines levels associated with clinical metabolic markers are strong predictors of the risk of developing IR and T2DM (12). Besides cytokines, the toll-like receptor 4 (TLR4) stimulus by its main ligand lipopolysaccharide (LPS) is also strongly associated with IR because TLR4 activation increases tumor necrosis factor alpha (TNFα) expression, which in turn impairs insulin signaling pathway in several tissues such as muscle and adipose tissue (13,14).

Although FNDC5/irisin levels and inflammatory markers have been related to glucose metabolism, the potential association between these markers is not yet established. Moreover, there are doubts whether higher or lower FNDC5/irisin levels are related to metabolic homeostasis, mainly in individuals with metabolic risk due to excess body fat. Based on these aspects, the aim of this study was to compare if higher or smaller FNDC5/ irisin levels are associated with inflammatory and metabolic markers, along with caloric/macronutrient intake, physical fitness and T2DM risk in obese middle- aged men with the absence of overt disease, through a cluster study, and also to correlate all variables analyzed with FNDC5/irisin.

## SUBJECTS AND METHODS

### Subjects

Inactive middle-aged (48,54 ± 5,91 years) male individuals, with body mass index (IMC: 31.01 ± 1.64 kg/m^2^), were recruited for the study through the local media.

Before the men's inclusion in the study, a complete medical examination was carried out, and individuals were excluded if they had an acute illness, severe hypertension, diabetes mellitus, myocardial infarction or orthopedic limitations. They should not be using any medication (as anti-diabetic, beta blocker, exogenous insulin, anti-inflammatory, thyroid hormone) that would interfere with the results. Participants could not be involved in regular exercise programs during the previous 12 months according to the Baecke Habitual Physical Activity Questionnaire and had to be insufficiently active according to the International Physical Activity Questionnaire (IPAQ) (15).

The individuals who met the criteria, and were approved at the initial medical evaluation, were assigned to the higher irisin group (HIG) and the smaller irisin group (SIG) after the cluster analysis was performed, as described in the Statistical analyses section. Twenty- two individuals matched the inclusion criteria, but two of them were excluded for not having all the required qualifications. Therefore, twenty individuals completed the study, and were divided into HIG (n = 11) and SIG (n = 9). The study protocol were explained before written consent was obtained. The study was in compliance with the Declaration of Helsinki and the procedures were previously approved by the Research Ethics Committee of the University of Campinas (appraisal number n° 1278/2011) and all subjects gave written informed consent before taking part.

### Experimental design

This is a cross-sectional study using a cluster approach. We evaluated cardiorespiratory fitness, muscle strength, resting metabolic rate (RMR), anthropometric parameters, body composition, feeding behavior, measurements of FNDC5/irisin, inflammatory markers and total cholesterol (TC), HDL-cholesterol (HDL), LDL-cholesterol (LDL), triglycerides (TG), insulin, glucose, glycated hemoglobin (HbA1c), homeostatic model assessment 2 beta, insulin sensibility and resistance (HOMA2B, S, IR), triglycerides and glucose index (TYG index), Quick index, systolic and diastolic blood pressure and diabetes mellitus type 2 index risk (T2DM index risk).

### Anthropometry and body fat

Body weight was measured with a calibrated manual scale (Filizola, São Paulo, Brazil) with a precision of 0.1 kg. Height was measured with a wall-mounted stadiometer, with a precision of 0.1 cm. BMI was calculated from the weight and height values. Neck (NC) and waist circumference (WC) were measured by the commonly established anatomical landmarks. Body density was estimated by the skinfold procedure with a skin-fold caliper (Lange, Beta Technology, Santa Cruz, CA, USA) at the chest, abdomen, thigh, triceps, subscapular, suprailiac, and mid-axillary points. The body fat percentage was obtained from body density with the Siri equation (16). The subcutaneous and visceral abdominal fat thickness was measured by the abdominal ultrasound method (17). All assessments were performed by the same professional.

### Blood sampling

Blood samples (~20 mL) were obtained from the antecubital vein in the morning (07.00 to 09.00), after 12h overnight fasting. All samples were divided into aliquots, processed immediately after collection and frozen at −80°C until later analysis. Serum samples were used for lipid profile and plasma (using EDTA antigulant) samples were used for irisin, glucose, insulin and HbA1c analysis.

### Lipid profile, glucose and insulin

Concentrations of TC, TG, HDL-C, and glucose were analyzed with an automatic analyzer (Technicon RA 1000 Chemistry Analyzer) and a commercially available kit (Laborlab, São Paulo, Brazil). The LDL-C was calculated according to the Friedewald equation (18). The insulin was determined by chemiluminescence with commercial kits (Elecsys insulin kit, Roche Diagnostics GmbH, Indianapolis, IN, USA) and an automatic biochemical analyzer (ARCHITECT i2000 SR, Abbot Diagnostics, IL, USA). The HbA1c was verified by high pressure liquid chromatography high performance (HPLC).

### FNDC5/irisin and adipokine measurements

FNDC5/irisin values were determined, duplicated by enzyme-linked immunosorbent assay (ELISA), according to the specifications of the manufacturer (Quantikine High-Sensitivity Kit (Lot n° L13112659), United States Biological, Swampscott, MA, USA). This is a polyclonal antibody kit, with capacity to identify FNDC5 complete protein and cleaved FNDC5 (Irisin). The kit also has the not cross-react with human peptides that have molecular patterns similar to FNDC5/Irisin as: fibronectin type III domain containing 4 (FNDC4), adiponectin, nicotinamide phosphoribosyltransferase (Nampt), retinol-binding protein 4 (RBP4), clusterin, leptin, vaspin, glutathione peroxidase 3 (GPX3), resistin, angiotensin-converting enzyme 2 (ACE2), lipocalin-2, angiopoietin-like protein3 (ANGPTL3), angiopoietin-like protein3 (ANGPTL6), delta and notch-like epidermal growth factor-related receptor (DNER), delta homolog 1 (DLK1), calreticulin and interleukin 33. The values are presented in micrograms per milliliter (ug/mL). The sample sensitivity was 1 nanogram per milliliter (ng/ml). The test range was 0.001-5 ug/ml. The values of all subjects, regardless of the group, are within range. The intra-assay and inter-assay kit coefficient and sensitivity were as follows: 4.86%, 8.02% and 1 ng/mL. All samples were measured in the same plates and collect in the same period.

Serum concentrations of resistin, leptin and adiponectin, TNF-α, interlukin 6 and interleukin 10 (IL6; IL10) were also determined by ELISA, following the specifications of the manufacturer (Quantikine High-Sensitivity Kit, R&D Systems, Minneapolis, MN, USA). The intra- and interassay coefficients and the sensitivity were as follows: TNF-α; 3.8%, 6.0% and 0.010 ng/mL; 7.4%, 6.5%, and 0.039 pg/mL for IL-6; 4.6%,7.8%, and 0.09 pg/mL for IL-10; 3.0%, 3.5% and 7.8 pg/mL for leptin; 2.8%, 5.9% and 0.246 ng/ml for adiponectin; 3.8%, 7.8% and 0.026 ng/mL for resistin.

To analyze LPS levels, plasma samples were diluted to 20% with endotoxin-free water and then heated to 70°C for 10 min to inactivate plasma proteins. Then serum LPS was quantified with a commercially available Limulus Amebocyte assay from Cambrex (Walkersville, MD, USA) according to the manufacturer's protocol. The samples were duplicated and the background subtracted.

### Blood pressure

Systolic and diastolic blood pressure assessments were performed after approximately 10 minutes of rest with a mercury sphygmomanometer and a stethoscope. The measurements were taken in the supine position by the same professional. All measurements were duplicated and the average of the two assessments was used.

### Formulas/Indexes

Beta-cell function, insulin sensibility and resistance were calculated respectively by the HOMA calculator using fasting concentrations of glucose and insulin equation (19).

Insulin resistance was also verified by the TYG index using the equation (fasting triglycerides (mg/dL) x fasting glucose (mg/dL)/2) (20). Insulin sensibility was evaluated using the QUICKI index by the following equation: Quicki = 1 ÷ (Log insulin + Log glicose) (21).

The risk of T2DM development was calculated with the algorithm for prediction of this disease in middle- aged adults in the Framingham Offspring study (22).

### Maximal-strength assessments

Maximal strength was measured by a one maximum repetition test (1RM) on bench press, leg press and arm curl exercises, performed on NakaGym equipment (São Paulo, Brazil). The 1RM tests were conducted as in Libardi and cols. (23). Before the beginning of the study, individuals performed two familiarization trials interleaved with 48h periods, to reduce the learning effects, as well as to establish the reproducibility of the tests in the exercise.

### Cardiorespiratory fitness test

The individuals performed a maximum-effort protocol on a Quinton TM55 treadmill (Bothell, WA, USA), where gas exchange data was collected continuously by means of an automated breath-by-breath metabolic cart (CPX; Medical Graphics, St Paul, MN, USA) (23).

### Rest metabolic rate (RMR)

The RMR was determined from oxygen consumption (O_2_) and carbon dioxide production (CO_2_) by indirect calorimetry of open circuit by the gas analysis system (CPX Ultima, MedGraphics, USA) and calculated in daily values (kcal/day) by the Weir equation (24).

The test was performed under laboratory conditions and after a fasting period of 12 hours. Volunteers used the gas analyzer connected to a facial mask, remaining silent in the supine position, avoiding movement and sleeping for 30 min (the initial 10 min were discarded), so that breath after breath could be obtained. The gas analyzer was calibrated before each test.

### Evaluation of caloric and macronutrient intake

Volunteers received diet records (DR) from trained nutritionists, who explained individually how to complete these records. Food scales were distributed and individuals were requested to list all food ingested during three-day food records to register intake on three different and non-consecutive days (two weekdays and one weekend day).

After completing the DR, the team of nutritionists met the volunteers so that if the data were not satisfactory consumer information could be compiled accurately. DRs were analyzed by Dietpro version 5i software. The estimation of nutrient intake was made with based in TACO/Unicamp table and USDA. The food intake assessment was derived from the average data of three recalls (25).

### Statistical analysis

Initially, using cluster analysis was performed (K-means cluster) with Statistica 6 software (StatSoft, USA) using the FNDC5/irisin level of each subject. This is an exploratory multi-variance analysis technique that allows classifying a set of data into homogeneous groups through similarities or dissimilarities between them. By “k means cluster” is possible to include the number of groups according to convenience. In function on the number of subjects we established two groups (higher and smaller irisin). After the establishment of the number of groups, the system assigned a centroid to each group. Subsequently each data (numerical object of the data set) has its Euclidean distance calculated with these centroids by means of a distance measure. The criterion for a data/numerical object to be allocated in a given group is its shortest distance from the centroid. After the cluster group's definition, data distribution was tested by the Shapiro-Wilk test. A Student's t-test was applied to analyze differences between clusters for parametric distributed variables and the Mann-Whitney U-test was used to compare the non-parametric distributed variables. We performed also correlations, using the Pearson's correlation between FNDC5/irisin for the parametric distributed variables and the Spearman's rank correlation to FNDC5/irisin for non-parametric distributed variables. Parametric distributed variables were expressed as mean ± standard deviation while non-parametric variables were expressed as median (Interquartile range). The p-value significance level was P ≤ 0.05 for all analyses.

## RESULTS

Table 1 presents anthropometric, body composition, caloric/macronutrient intake, physical fitness and RMR values of Higher Irisin Group (HIG) and Smaller Irisin Group (SIG). The hallmark results were that HIG had smaller weight (p = 0.04), neck circumference (p = 0.02) and lipid intake (p = 0.05).

**Table 1:** Anthropometric markers, body fat, caloric/macronutrient intake, physical fitness and rest metabolic rate of groups higher and smaller irisin

Variable	Higher Irisin Group (HIG) n = 11	Smaller Irisin Group (SIG) n = 9
Age (years)	49.09 (45.33-52.84)	48 (43.20-52.80)
Weight (kg)	90.16 ± 6.66	96.68 ± 7.02*
Height (m)	1.72 ± 0.06	1.75 ± 0.05
BMI (kg/m^2^)	30.63 ± 1.68	31.47 ± 1.57
Body fat (%)	35.47 ± 5.43	34.05 ± 4.59
Waist circumference (cm)	101.1 ± 5.2	102.7 ± 4.7
Neck circumference (cm)	40.88 ± 1.56	42.87 ± 1.99*
US SF (mm)	26.1 ± 8.7	24.76 ± 6.10
US VF (mm)	65.46 ± 14.33	68.32 ± 19.43
Carbohydrates (g/day)	257 ± 56	276 ± 90
Lipids (g/day)	76.10 ± 22	100.59 ± 32*
Proteins (g/day)	99.5 ± 6	107.8 ± 27.5
Caloric Intake (kcal)	2154 ± 471	2515±695
1 RM leg press (kg)	293 ± 84.88	302 ± 56.4
1RM bench press (kg)	70.5 ± 15.79	70.14 ± 15.81
1RM arm curl (kg)	29.18 (26.16-32.19)	30.57 (26.62-34.51)
VO2max. (mL/kg^-1^/min^-1^)	26.84 ± 3.64	25.95 ± 4.04
RMR (kcal)	1331 ± 200.82	1397 ± 235.52

BMI: body mass index; US SF: ultrasound subcutaneous fat; US VF: ultrasound visceral fat; RM: maximum repetition; VO_2_max.: maximum volume of oxygen; RMR: Rest metabolic rate.

* Significant difference HIG and SIG (p < 0.05). Parametric variables were expressed as the mean ± standard deviation; non-parametric variables were expressed as the median (Interquartile range).

Table 2 shows the glycemic homeostasis markers, lipid profile and systolic/diastolic blood pressure values of both groups. HIG compared to SIG presented smaller insulin (p = 0.02), triglyceride levels (p = 0.01), and insulin resistance by the HOMA-IR (p = 0.01), along with TYG index (p = 0.02). HIG still had better insulin sensibility according to HOMA-S (p = 0.01) and QUICKI index (p < 0.01). There was also a tendency of HIG to exhibit smaller VLDL levels (p = 0.07).

**Table 2:** Biochemical, metabolic indexes and hemodynamic markers of groups higher and smaller Irisin

Variable	Higher Irisin Group (HIG) n = 11	Smaller Irisin Group (SIG) n = 9
Fasting insulin (uU/mL)	9.14 ± 2.09	14.26 ± 5.9*
Fasting glucose (mmol/l)	5.18 ± 0.25	5.24 ± 0.72
Hb_A1c:_ (%)	5.34 (5.15-5.53)	5.35 (4.79-5.91)
HOMA2-B	107.03 (90.93-123.13)	143.03 (87.66-198.39)
HOMA2-S	83.48 ± 23.7	58.16 ± 19.24*
HOMA2-IR	1.23 ± 0.27	1.81 ± 0.71*
QUICKI Index	0.34 ± 0.02	0.31 ± 0.01*
TYG Index	4.69 ± 0.19	4.87 ± 0.21*
Triglycerides (mmol/l)	1.43 9 (1.09-1.81)	2.27 (1.60-2.94)*
Total Cholesterol (mmol/l)	4.64 ± 0.90	5.22 ± 1.15
HDL (mmol/l)	1.05 ± (0.94-1.59)	1.09 (0.87-1.31)
VLDL (mmol/l)	0.72 ± 0.33	1.04 ± 0.40†
LDL (mmol/l)	2.86 ± 0.76	3.02 ± 1.04
Systolic pressure (mm/Hg)	125.82 ± 16.26	117.14 ± 12.44
Diastolic pressure (mm/Hg)	87.09 ± 11.95	80.28 ± 5.6

Hb_A1c_: glycated hemoglobin; HOMA-B: homeostatic model assessment - beta; HOMA-S: homeostatic model assessment - sensibility; HOMA-IR: homeostatic model assessment - insulin resistance; TYG Index: triglycerides/glucose index; HDL: high density lipoprotein; VLDL: very low density lipoprotein; LDL: low density lipoprotein.

* significant difference between HIG and SIG (p < 0.05).

† trend difference between HIG and SIG (p between 0.051 and 0.090). Parametric variables were expressed as the mean ± standard deviation; non-parametric variables were expressed as the median (interquartile range).

**Figure 1:**
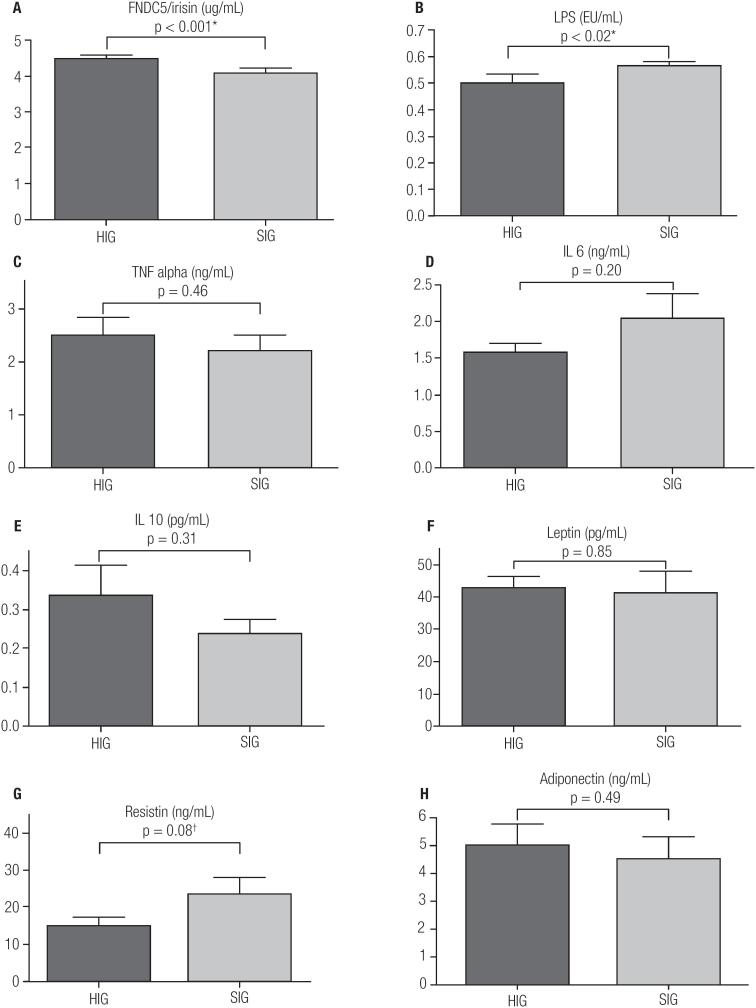
FNDC5/irisin levels and inflammatory markers of groups higher and smaller irisin. HIG and SIG levels of **A:** FNDC5/irisin. **B:** LPS (lipopolysaccharide). **C:** TNF alpha (tumor necrosis factor alpha). **D:** IL6 (interleukin 6). **E:** IL10 (interleukin 10). **F:** leptin. **G:** Resistin. **H:** adiponectin. *: significant difference between HIG and SIG (p < 0.05). †: Trend difference between HIG and SIG (p between 0.051 and 0.09).

The FNDC5/irisin, LPS, cytokines and adipokine levels are presented in Figure 1 A-H. Besides higher FNDC5/irisin levels in HIG (HIG 4.46 ± 0.10 x SIG 4.07 ± 0.14, p < 0.01), this group exhibited smaller LPS levels (HIG 0.50 ± 0.05 x SIG 0.56 ± 0.06, p = 0.02) and tendency to lower levels of resistin (HIG 14.88 ± 8.21 x SIG 23.65 ± 12.93, p = 0.08).

**Figure 2:**
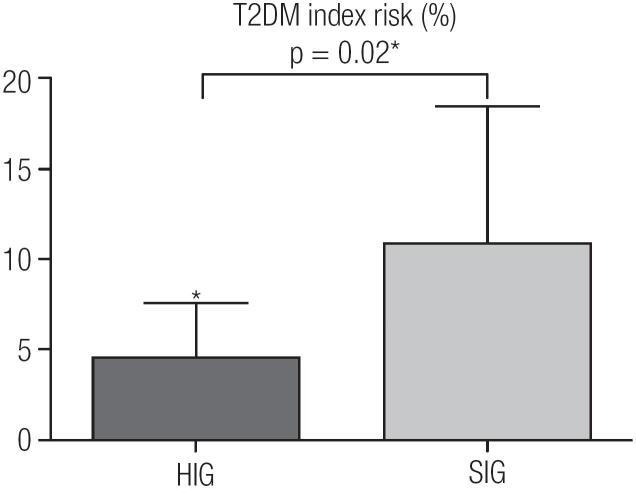
Diabetes mellitus type 2 index risk of groups higher and smaller Irisin. T2DM: diabetes mellitus type 2. * Significant difference between HIG and SIG (p < 0.05).

Proving the better metabolic status in HIG, this group still showed a smaller risk of developing T2DM (HIG 4.55 ± 3.01 x SIG 10.88 ± 9.82, p = 0.02) (Figure 2).

About the correlations, there are significant inverse correlations between FNDC5/irisin and body weight (r −0.46, p = 0.04), neck circumference (r −0.51, p = 0.02), free fat mass (r −0.49, p = 0.02), triglycerides (r −0.43, p = 0.05) and risk of developing T2DM (r −0.61, p = 0.04).

## DISCUSSION

Herein, using a cluster study, our results show that obese middle-aged men with higher irisin levels have a better metabolic profile along with lower risk of T2DM development and LPS levels. Furthermore some anthropometrics/body composition variables and risk of T2DM are inversely related to FNDC5/irisin levels.

Interestingly, the better clinical parameters observed were associated with high irisin levels, differently of other studies that associated lower FNDC5/irisin levels to healthy state (9-11). Our results also go against works that put in doubt the FNDC5/irisin beneficial effects (26). Thus, the present study corroborate clinically with early studies in animals and in vitro (1,2) that associated FNDC5/irisin stimulus with a metabolic improvement in humans.

The FNDC5/irisin levels have been reason of several questionings and doubts. Initially higher serum levels were associated with a possible FNDC5/irisin resistance, as occur with insulin (11), however our results showed that this fact did not occur because the HIG showed smaller insulin resistance. Some authors have suggested a possible “irisinemia” as a compensatory effect of organism to try maintain the metabolic homeostasis by irisin secretion (27). However, the better hypothesis must be the negative influence of hyperglycemia and serum lipids on FNDC5/irisin levels, since free fatty acid and glucose *in vitro* stimulation decrease expression/secretion of these peptides (28). Our metabolic results associated a cluster of FNDC5/irisin levels bring similar clinical evidence of this in-vitro study, and consolidate the studies that observed lower levels of FNDC5/irisin correlated with dysfunctions and metabolic diseases (6,8,29,30).

Studies that analyzed the relation of inflammatory markers and FNDC5/irisin levels are scarce in the literature and the present work show for the first time evidences about this context. Given the metabolic profile results, it would be expected that HIG exhibited better levels of global inflammatory markers, but this is partially evidenced through results observed for LPS and the trend towards reduction of circulating resistin (p = 0.08).

LPS is an endotoxin related to insulin resistance, once it binds TLR4 and triggers intracellular inflammatory responses (14). The exact relation of FNDC5/irisin and LPS levels is something that needs to be better understood, however, some hypotheses may be raised, such as the LPS interfering negatively on FNDC5/ irisin secretion as observed in other study when glucose and free fat acid stimulus *in vitro* down-regulation FNDC5/irisin secretion. On the other hand, FNDC5/ irisin has been associated with endothelial integrity (31), and vascular disease and injury can influence LPS gut permeability and systemic inflammation (32).

Interestingly, a recent study showed that LPS is a negative regulator of adipose tissue browning process (33). However, this work not analyzed the relation between LPS and FNDC5/irisin (33). Our present results seem to indicate that this negative LPS influence in browning process could also involve a decrease of FNDC5/irisin, one of the main stimulator of these process. Reinforce this idea the higher fat intake in SIG because the increase of fat, specially the saturated type, is related to LPS absorption, thus the LPS increase could be an inhibitor of FNDC5/ irisin, as observed by excess of glucose and free fat acids (28), which could explain the LPS as a negative regulator of browning (33). Previous study found a positive correlation between FNDC5/irisin levels and carbohydrate intake (34), indicated, together with our result that FNDC5/irisin and macronutrients might have an important interaction that certainly needs to be deeply addressed in future researches, as well as better analyze the relationship between FNDC5/irisin and LPS.

The tendency of lower resistin level is another positive HIG result, because the increase in this adipokine is related to the insulin resistance and worse inflammatory condition (12). Also, it is not possible to explain the exact relation between FNDC5/irisin and resistin levels. New studies could be to focus also on these markers relations.

Even for adipokine results, although leptin could be a negative regulator of FNDC5/irisin (3), our results demonstrate that, at least in serum obese men, an interrelationship between these biomarkers does not seem to occur. Similar to leptin, TNFα, adiponectin, IL 6 and IL 10 serum are not related to higher or smaller FNDC5/irisin levels. However, molecular relationships need to be further investigated.

All of the actions assigned to FNDC5/irisin seems to stimulate the increase of lipid substrate consumption by mitochondria (1). Based in the FNDC5/irisin effects on energy expenditure increase observed in animals and *in vitro* studies (1,2), and also in the better metabolic profile verified in HIG, we could expect a difference in RMR between HIG and SIG. However, our results did not show any significant difference in the RMR between two clusters groups. Indeed, such result is in line with previous studies (35,36), which also not observed the relation between FNDC5/irisin levels and rest energy expenditure in humans. The methodology for evaluating the rest energy expenditure in the current study has been validated previously (24), but it is a mix of direct and indirect tests, and perhaps the use of only direct methods or tests to evaluate the percentage of energy substrates used during rest would results in significant difference between groups, because several factors can interfere in that variable.

The significant lowest levels of triglycerides and downtrend in VLDL in HIG observed in the present study corroborate with other study (30), which observed that higher levels of lipids in blood and liver have correlation with lower FNDC5/irisin levels.

About correlations, were observed inverse associations between FNDC5/irisin and body weight, free fat mass, neck circumference, triglycerides and risk of developing T2DM risk results, strengthening the others find of present work. The body weight, free fat mass and triglycerides have been related to FNDC/ irisin (5,30,37), indicating that body composition, especially body fat, and serum lipids could has influence about levels of this peptides, however, it is important to mention that these correlations presented a fragile “r” values. Although several metabolic glycemic control variables has been associated to FNDC5/ irisin levels, to our knowledge, it is the first time that neck circumference (a practice factor related to insulin resistance) (38) and a developing risk T2DM global index are inversely related to FNDC5/irisin levels, indicating, once again, that in humans, the levels of this peptides is fully combined with insulin resistance and glucose tolerance, as observed in animals and *in vitro* studies (1,39). Based on insulin sensibility and resistance observed between groups it would be expected that correlation with others markers of glucose homeostasis were observed besides neck circumference, however, it is important to cite that there are trend statistic between FNDC5/irisin and HOMA-S (p = 0.07), Quicki index (p = 0.08) and TYG index (p = 0.07), what indicate that results go in this direction, perhaps in a higher sample of subjects these results could be significant.

The HIG showed higher body weight, however this result should not be an intervening factor in the results, because both cluster groups have similar BMI, fat mass, and waist circumference, parameters that can interfere more significantly in FNDC5/irisin levels than body weight analyzed in isolation (5). Moreover, the difference between the body weight of the groups may be related to the average height difference among them (HIG 1.72 m x SIG 1.75 m) and BMI similarity between groups prove this idea, being this analyze more applicable because the weight must be related to the height.

FNDC5/irisin levels difference average between HIG and SIG is approximately 10%. Two points can support this difference as relevant. First, the difference between fasting glucose levels of diabetic subject (126 mg/dL) and the normoglycemia (99 mg/dL) is around 20%. Second, a previous study has demonstrated a difference about 30% in irisin levels, when comparing type 2 diabetic and health subjects (7). Together, these data strongly support the idea that in non-diabetic people, i.e., insulin resistant people, such 10% difference in irisin levels is really significant.

Physical exercise and temperature was reported having influence on FNDC5/irisin levels (39). However, these factors should not have interfered in present results, once both groups underwent the experiment during similar periods; the region where the research was conducted did not present large thermal fluctuations or seasons with extreme temperatures; all subjects were not engage in regular exercise programs during the previous 12 months and were classified as insufficiently active according to the Baecke Habitual Physical Activity Questionnaire and International Physical Activity Questionnaire (IPAQ) (15).

The present work is a cross-sectional study, with a relatively limited sample size of obese individuals, and did not investigate the relationship of cause and effect for the results found. However, it is important to emphasize that these group analyzed is extremely homogeneous and were selected by strict inclusion criteria. Moreover, the present study analyzed more than 40 clinical variables, to our knowledge, are scarce in literature human's studies with FNDC5/irisn and these significant numbers of variables investigated. Even with a small sample, the evidence here presented confirms the FNDC5/irisin positive relation with metabolic homeostasis in humans, particularly in obese men. Based in the relation of smaller FNDC5/irisin with worse metabolic state, these peptides may, in the future, be a marker for the presence of metabolic diseases and also a therapeutic target. However, the ideal FNDC5/irisin values are not known, so further work should focus on this aim.

In conclusion, higher FNDC5/irisin levels in grade 1 obese men are related to a better metabolic profile, less risk of developing T2DM, decrease of serum LPS.

Epidemiological studies with a largest number of subjects must be performed to confirm the present results and to establish cut-off points for optimal FNDC5/irisin levels and also for this peptide to be used as a metabolic risk marker. To evaluate a possible and exact relationship between FNDC5írisin and LPS, other works should also be designed. Lastly, how physical exercise are one of the main stimulators of FNDC5/ irisin secretion, studies comparing the exercise effects on these peptides level in groups of high and small irisin levels also must be considered.
